# Servant leadership and project success: the mediating roles of team learning orientation and team agility

**DOI:** 10.3389/fpsyg.2024.1417604

**Published:** 2024-08-01

**Authors:** Huibin Han, Xiaojia Zhang

**Affiliations:** ^1^School of Economics and Management, Liaoning University of Technology, Jinzhou, Liaoning, China; ^2^SolBridge International School of Business, Woosong University, Daejeon, Republic of Korea

**Keywords:** servant leadership, team learning orientation, team agility, project success, project management

## Abstract

Drawing from social learning theory, this study aims to explore the mediating effects of team learning orientation and team agility on the relationship between servant leadership and project success in the context of construction projects. Based on data collected from 306 construction project members in China, the findings reveal that servant leadership exerts a positive influence on project success. Additionally, servant leadership significantly enhances both team learning orientation and team agility, which in turn contribute to project success. Furthermore, the results demonstrate the serial and parallel mediating roles of team learning orientation and team agility between servant leadership and project success. Theoretical and practical implications were also provided based on the findings.

## 1 Introduction

Traditionally, projects have been viewed as merely technical systems with an emphasis placed on employing sophisticated methodologies and tools (Imam and Zaheer, 2021). However, despite such techniques, numerous projects have continued encountering failures (Ellahi et al., 2022). The primary causes of project failures in China are often associated with management issues rather than technical aspects (Zhou et al., 2019). An evolving perspective in the project management literature has brought attention to the significant influence of human behavior and dynamics as pivotal success factors, rather than just technical aspects (Jugdev and Müller, 2005). This shifting focus underscores leadership’s vital role, with studies showing 80% of project failures attributed to ineffective leadership (Fareed et al., 2023). Accordingly, different leadership styles have been increasingly investigated on project success (PS), including transformational leadership (Aga et al., 2016), ethical leadership (Bhatti et al., 2021), servant leadership (Ellahi et al., 2022), and shared leadership (Imam and Zaheer, 2021).

Among the leadership styles, servant leadership (SL), characterized by its focus on people, holds particular potential for project contexts marked by complexity and uncertainty. Existing studies support that leaders centered on people tend to exhibit greater effectiveness in ensuring the successful delivery of projects (Thamhain, 2004; Behrendt et al., 2017), including the specific context of China (Chen and Tjosvold, 2014). In addition, SL has been linked to favorable outcomes, including intrinsic motivation (Xue et al., 2022), work engagement (Bao et al., 2018), and emotional intelligence (Miao et al., 2021). These elements are empirically supported to contribute to PS (Ellahi et al., 2022; Malik et al., 2022). In the meta-analysis by Lee et al. (2020), SL shows incremental validity compared to other leadership styles like authentic, ethical, and transformational leadership. Thus, SL acts as an effective leadership style in the context of project-based organizations characterized by complexity and uncertainty.

There is a growing focus on how SL contributes to PS within the project management literature (Bilal et al., 2020; Nauman et al., 2022a). SL is demonstrated to impact PS directly and indirectly. Specifically, some mediators between SL and PS have been identified by previous studies, including work engagement and project work withdrawal (Nauman et al., 2022b), emotional intelligence and job stress (Malik et al., 2022), team motivation and team effectiveness (Ellahi et al., 2022). However, research gaps remain in incorporating team-level process variables like team learning orientation (TLO) and team agility (TA) into the research framework and exploring their mediating mechanisms in the relationship between SL and PS. Malik et al. (2022) suggest that more studies need to explore the mediating mechanisms between SL and PS. Similarly, Nauman et al. (2022a) suggest that future work could incorporate intervening variables in this relationship. To respond to these calls, this study posits that TLO and TA serve as mediators between SL and PS. Exploring TLO and TA underlying SL and PS holds significance for the following reasons.

The swift environmental changes present both challenges and chances for successfully managing projects (Ali et al., 2021). For example, the environments surrounding construction projects, both internal and external, tend to be changing and not stable (Love et al., 2002). The increasing dynamism necessitates work teams to proactively engage in continuous learning and self-improvement to effectively respond to changes (Pearsall and Venkataramani, 2015). TLO, marked by a shared understanding that values active learning, serves as a critical mechanism, motivating members to participate in learning behaviors (Chiu et al., 2021). It is worth noting that TLO significantly influences positive team processes, including team task reflexivity (Wang and Lei, 2018), team planning processes (Pearsall and Venkataramani, 2015), and adaptive behaviors (Bunderson and Sutcliffe, 2003), all of which are essential for PS. Moreover, leadership has been identified as an effective predictor of learning orientation (Coad and Berry, 1998). As a result, by exploring the mediating role of TLO between SL and PS, this study aims to offer deeper insights into the mechanisms through which SL impacts PS.

In addition, TA is another effective response to the rapidly changing environment (Krüger, 2023). TA assists teams in swiftly adapting to uncertainties during projects (Conforto et al., 2014), constituting a fundamental component for long-lasting success (Denning, 2013). Empirical studies have connected TA to constructive team outcomes like performance (Liu et al., 2015) and shared mental models (Krüger, 2023). Moreover, academics have explored agility determinants, identifying leadership as a potent one (Akkaya and Tabak, 2020). Thus, by investigating the mediating role of TA between SL and PS, this study provides a better understanding of how to effectively leverage the influence of SL in dynamic environments to achieve PS.

Furthermore, the complex and dynamic nature of projects implies that TLO and TA may serially mediate the link between SL and PS. As noted by Hayes (2018), investigating serial mediation is critically important for delineating the distinct effects of causation. Servant leaders prioritize the needs of subordinates and facilitate subordinates’ growth to their full potential (Graham, 1991). Moreover, servant leaders focus on stewardship motivates teams to question old assumptions and seek new knowledge (Yoshida et al., 2014). These processes nurture TLO which focuses on acquiring new skills and knowledge. As noted by Edmondson (1999), team learning behaviors fostered team flexibility which is the prerequisite for TA. Ultimately, despite uncertainty, TA to adapt and respond to changes enhances team adaptation, which in turn helps the project reach its objectives (Vázquez-Bustelo et al., 2007). Examining this serial mediation of TLO to TA between SL and PS will provide nuanced understanding of how servant leaders can translate their impact and improve project delivery.

**Figure 1:**
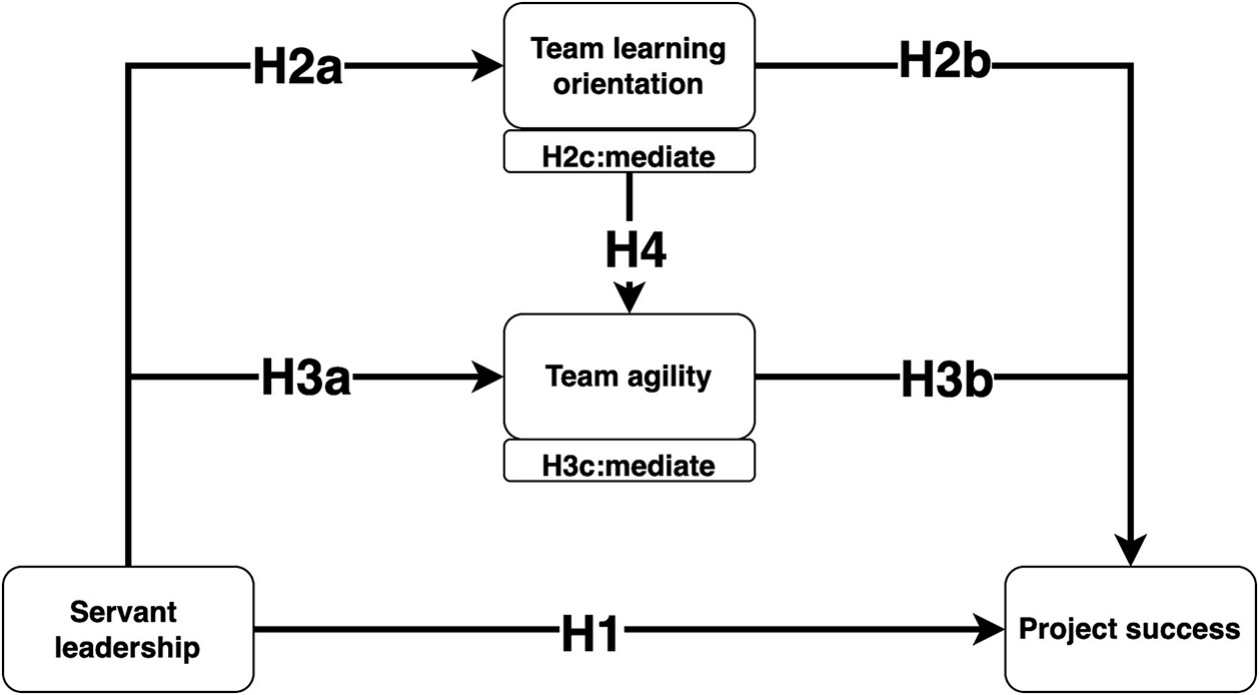
Conceptual Framework.

To address these questions, drawn from social learning theory (SLT). This study employed Structural Equation Modeling (SEM) to analyze the correlation among the variables based on a survey of 306 construction project members in China. The data gathered from this survey will be analyzed to investigate the proposed model, as depicted in Figure 1. The paper aims to offer substantial practical insights and contribute valuable theoretical perspectives on the mechanisms through which SL exerts its influence on PS.

## 2 Theoretical underpinning

The SLT was first proposed by Albert Bandura in the 1960s and 1970s (Bandura and Walters, 1977). This theory posits that human behavior is largely learned through observation, modeling, and vicarious reinforcement (Bandura, 1999). It highlights that individuals can learn new behaviors by observing others with a process termed observational learning or modeling (Liden et al., 2014). Through this process of observation and modeling, individuals can anticipate the potential outcomes of their own actions in similar situations, thereby adjusting and regulating their behaviors accordingly (Davis and Luthans, 1980).

Drawing from SLT, servant leaders act as role models for followers to observe and emulate (Sendjaya et al., 2008). This modeling effect can be particularly powerful in shaping organizational team climates, norms, and practices (Argote, 2011). For example, the modeling influence of servant leaders can foster positive knowledge-sharing climates and service climates (Hunter et al., 2013; Song et al., 2015), creating a beneficial team environment for team learning. Moreover, the influences that servant leaders exert on followers manifest collectively and iteratively, subsequently stimulating positive changes within teams (Russell and Gregory Stone, 2002). In addition, SLT consistently finds support for the modeling of behavior, both through laboratory experiments and practical applications, regarding the influence of leaders’ behaviors on their subordinates’ (Eva et al., 2019; Nauman et al., 2022a). Therefore, SLT provides a useful theoretical underpinning for understanding how servant leaders can model desired behaviors and competencies to facilitate team performance.

## 3 Literature review

### 3.1 Servant leadership

The expression “servant leadership” was originally developed by Greenleaf (1970), and SL has garnered increasing attention from scholars in recent years (Gardner et al., 2020). Servant leaders refer to leaders who “place the needs of their subordinates before their own needs and center their efforts on helping subordinates grow to reach their maximum potential and achieve optimal organizational and career success” (Liden et al., 2008, p. 163). Different from traditional leadership styles that highlight the leader’s power and authority, SL emphasizes the leader’s responsibility to serve by prioritizing the requirements of subordinates (Bilal et al., 2020).

Servant leadership is different from other value-based leaderships (Schowalter and Volmer, 2023). Unlike transformational leadership, which prioritizes organizational goals over followers’ needs, SL accentuates fulfilling the psychological necessities of subordinates with greater weight, designating it as a principal objective (van Dierendonck et al., 2014). In addition, SL is characterized by a propensity for altruistic behavior, driven by the motive to serve others, rather than solely focusing on being authentic in interpersonal interactions like authentic leadership (Eva et al., 2019). Furthermore, comparing to ethical leadership, where leaders typically influence followers to be ethically conscientious and act morally (Ko et al., 2018), servant leaders provide more attention to specific directions for followers, an aspect that is relatively absent in the approach of ethical leaders.

In an analytical review conducted by Eva et al. (2019), they critically examined 16 extant instruments assessing SL, evaluating their scale development and validation. The measurement tool called SLBS-6 developed by Sendjaya et al. (2019) emerged as noteworthy for its meticulous construction and validation processes. SLBS-6 authentically reflects the conceptualization of Greenleaf (1970) and Graham (1991) that spirituality is the core of SL, and followers are impacted by leaders’ humility (Eva et al., 2019). Moreover, recent empirical studies have also confirmed that the SLBS-6 instrument has demonstrated satisfactory psychometric properties in terms of reliability and validity (Khan et al., 2022). Furthermore, the original SLBS-35 developed by Sendjaya et al. (2008) is demonstrated that the multiple dimensions of this measurement are most accurately viewed as one higher construct (Sendjaya and Cooper, 2011). Thus, given that this study examines the overall effect of SL without distinguishing between dimensions, SLBS-6 has been adopted as the measurement for SL.

Extant empirical research has substantiated the positive connection between SL and anticipated outcomes such as enhanced performance and organizational citizenship behaviors (Schowalter and Volmer, 2023). Moreover, accumulating evidence suggests that SL nurtures antecedent conditions conducive to PS, including fostering project identification (Nauman et al., 2022b), cultivating a collaborative culture (Nauman et al., 2022a), and bolstering team motivation (Ellahi et al., 2022). Consequently, SL appears particularly well-suited for project-based organizational contexts. However, the specific mechanisms through which SL is translated into improved project outcomes require further examination.

### 3.2 Team learning orientation

In recent decades, scholars have extensively delved into the exploration of learning orientation (Hakala, 2011; Gemici and Zehir, 2021). Learning orientation refers to “a concern for, and dedication to, developing one’s competence” (Gong et al., 2009, p. 765). It stands as a critical foundation for nurturing learning competence, a trait that is prominently displayed and interwoven across various organizational levels, including both individuals and collectives. Notably, Senge (1990) posits that teams, instead of individuals, represent the basic component of learning within organizations. Moreover, Khedhaouria et al. (2017) also underscored the presence of team learning and emphasized the crucial need for exploration at the team level.

The TLO refers to “an emergent group climate characterized by team members’ shared understanding that continual learning and self-development is an essential team objective” (Chiu et al., 2021, p. 190). It plays a crucial role in determining team members’ learning behaviors (Edmondson, 1999). Teams that do not engage in appropriate learning activities tend to be less effective at both individual and team levels of performance (Savelsbergh et al., 2012). TLO lies in its ability to motivate members to undertake various learning actions, thereby facilitating team adaptability and effectiveness (Chiu et al., 2021).

Social learning theory emphasizes the procedure by which individuals obtain knowledge and skills by observing, imitating, and interacting with others (Bandura and Walters, 1977). The conducive climate fostered by TLO facilitates this learning process effectively (Chiu et al., 2021). Thus, the interactions within teams are expected to yield favorable outcomes. Empirical studies have also consistently demonstrated that TLO represents a robust antecedent of positive team behaviors, including task reflexivity (Wang and Lei, 2018), team planning processes (Pearsall and Venkataramani, 2015), and adaptive behaviors (Bunderson and Sutcliffe, 2003). Additionally, scholars have claimed that the development of efficient intra-project learning can advance project-based organizations’ competitiveness (Jugdev and Mathur, 2013). Nevertheless, the exploration of antecedents for TLO remains limited.

### 3.3 Team agility

Team agility originated from software development to improve handling of changing requirements, productivity, and business alignment (Brown and Eisenhardt, 1995; Campanelli and Parreiras, 2015). TA is defined as “a team’s ability to respond to unpredictable changes in proper ways and to take advantage of these changes as opportunities” (Liu et al., 2015, p. 297). It represents the manifestation of agility at the team level, enabling organizations to translate their agile capabilities into action. Research shows that agile teams respond to change, take action, and make decisions more quickly than traditional teams, and TA is regarded as an emerging pillar of project management that can enable sustained success (Krüger, 2023).

Although TA originated from the software development domain, its underlying principles and practices have demonstrated potential applicability across various project-based industries and contexts beyond software. Several studies have explored the adoption of agile methodologies in non-software projects, such as construction (Loforte Ribeiro and Timóteo Fernandes, 2010; Kashikar et al., 2016), product development (Lill and Wald, 2021), and marketing (Kalaignanam et al., 2021). The core tenets of TA, including responsiveness to change, customer collaboration, iterative delivery, and self-organization, can be valuable in any project environment characterized by uncertainty, complexity, and evolving requirements (Conforto et al., 2014). Notably, the uncertain and dynamic nature of construction projects aligns well with the strengths of TA, making it a potentially suitable approach for addressing the unpredictable conditions inherent in such project environments (Layton et al., 2020).

In addition, TA plays a crucial role in fostering PS. The notion of TA derives from the agile principles and values outlined in the Agile Manifesto (Beck et al., 2001). The Agile Manifesto emphasizes the primacy of interactions over processes and tools, as well as the necessity of responding to change rather than rigidly adhering to predetermined plans. These core values underscore the importance of teamwork and flexibility, which are fundamental elements that can facilitate the achievement of successful project outcomes (Zaman et al., 2019; Ali et al., 2021). Moreover, agile practices have been demonstrated to enhance trust and teamwork among team members, rendering them particularly well-suited for complex, uncertain projects characterized by evolving requirements (McHugh et al., 2012; Qureshi et al., 2014). By embracing the principles of TA, project teams can cultivate an environment that promotes adaptability, collaboration, and continuous improvement, thereby increasing their capacity to navigate uncertainties, respond to changes, and ultimately contribute to the realization of PS.

Since TA is a relatively new approach for project teams (Conforto et al., 2016), studies on TA remain limited. Scholars have explored organizational agility antecedents, identifying leadership as an influential factor (Akkaya and Tabak, 2020; AlNuaimi et al., 2022). Additionally, empirical studies have linked TA to positive team outcomes like performance (Liu et al., 2015) and shared mental models (Krüger, 2023). Based on these findings, this study argues that TA could potentially act as a mediator between SL and PS.

### 3.4 Project success

Project success was originally defined as completing a project within the expected schedule, budget, and quality (Atkinson, 1999). However, PS’s definition has evolved over time, with different organizations and scholars using varying criteria. The Project Management Institute (PMI) expanded the definition to include meeting stakeholders’ diverse concerns and expectations (Project Management Institute, 2000). While the British Association for Project Management stated that satisfying stakeholders’ needs should be included in PS (Fareed and Su, 2022). Academically, Ika (2009) indicated that achieving strategic objectives and sponsor satisfaction are two critical factors for PS. Joslin and Müller (2015) argue that the anticipated project outcome should also be included with the definition of PS.

Although a consensus definition remains elusive, understanding of PS has broadened from the traditional constraints of schedule, budget and quality to a multifaceted success incorporating diverse perspectives (Pollack et al., 2018). The review of Joslin and Müller (2015) found that Pinto and colleagues’ frameworks most comprehensive for measuring PS. Aga et al. (2016) later adapted and expanded this measurement. Thus, based on the study from Aga et al. (2016), this study employs the composite measurement to assess PS.

## 4 Hypothesis development

### 4.1 SL and PS

Social learning theory posits that followers learn behaviors by perceiving and copying role models (Bandura and Walters, 1977). In the context of projects, the leader acts as a salient role model for team members. Servant leaders prioritize their teams’ growth and wellbeing over personal interests (Eva et al., 2019). By showing voluntary subordination, responsible morality, and transforming influence, servant leaders demonstrate service-oriented conduct (Sendjaya et al., 2019). Project teams observe and internalize similar servant leader behaviors. They become more motivated to emulate the altruism, kindness, and community stewardship exhibited by their leader (Krog and Govender, 2015). Via the procedure of social learning, servant leaders shape team dynamics to be more collaborative and committed to shared goals. As supported by Raziq et al. (2018) and Nauman et al. (2022a), collaboration culture and goal clarity have been examined to be effect predictors for PS. In addition, SL has been recognized as a determinant of positive outcomes. Ruiz-Palomino et al. (2023) found that SL improves team performance. Moreover, both Malik et al. (2022) and Nauman et al. (2022b) have provided evidence that SL has a positive effect on PS. Thus, the hypothesis is suggested:

*H1*: SL positively influences PS.

### 4.2 SL and TLO

According to SLT, the behaviors exhibited by leaders impact and motivates subordinates’ actions, promoting the emulations of similar behavior across the organizational hierarchy (Mayer et al., 2012). SL demonstrates that the central role of a leader is to serve the subordinates (Nauman et al., 2022b). They prioritize assisting the needs of subordinates by open communication and transcend their own interests to facilitate subordinates’ growth to their potential (Graham, 1991). By modeling openness to feedback, reflection on failures, and striving for self-betterment, servant leaders demonstrate a group climate with continual learning and self-development. Moreover, servant leaders focus on stewardship motivates teams to question old assumptions and seek new knowledge (Yoshida et al., 2014). In this scenario, team members will gradually develop learning-focused behaviors. Their emphasis on growth and reflection establishes norms that learning is valuable. As a result, SL fosters an environment optimized for continuous team learning and development. A number of studies recommend that group climate where learning is emphasized can nurture TLO (Bunderson and Sutcliffe, 2003). In addition, SL has shown a positive connection with organizational learning (Goestjahjanti et al., 2022) and team-based learning (Grobler and Flotman, 2021). Thus, the hypothesis is proposed:

*H2a*: SL positively influences TLO.

### 4.3 TLO and PS

The TLO decides the extent and significance of members’ learning behaviors (Edmondson, 1999). A high level learning orientation motivates engagement in uncovering others’ interests and developing plans to optimize collective performance (Pearsall and Venkataramani, 2015). When members observe their colleagues actively engaging in learning processes and accept exploration as valued group norms, they become more likely to pursue novel endeavors (Rosenthal and Zimmerman, 2014). TLO also enables adaptation to changing project-based environments through continual work process optimization and outcome improvements (Wang and Lei, 2018). Moreover, research indicates TLO positively impacts team reflexivity and performance (Wang and Lei, 2018), goal mental models and planning processes (Pearsall and Venkataramani, 2015). Thus, the hypothesis is suggested:

*H2b*: TLO positively influences PS.

### 4.4 The mediating role TLO

Servant leaders exhibit openness to learning, a willingness to admit mistakes, and a focus on the collective interest (Eva et al., 2019). According to SLT (Bandura and Walters, 1977), project members emulate these learning-focused behaviors from servant leaders. Additionally, servant leaders cultivate a learning-oriented climate by questioning old assumptions and seeking new knowledge (Yoshida et al., 2014). Members engaged in this process recognize the value and importance of learning. TLO emerges when all team members collectively value, seek out, and reflect on knowledge, skills, and feedback to enhance team performance (Bunderson and Sutcliffe, 2003). Consequently, TLO encourages behaviors such as information sharing, seeking help, expressing concerns, and reflecting on processes. These behaviors enable teams to identify problems, build knowledge, and improve (Edmondson, 1999). By enhancing team knowledge, coordination, and performance, TLO contributes to superior project outcomes and success. Therefore, the hypothesis is suggested:

*H2c*: TLO plays a positive mediating role between SL and PS.

### 4.5 SL and TA

The SL may enhance TA through the provision of followers’ empowerment and autonomy. Servant leaders authorize their followers by delegating significant obligations, granting them to cope with situations autonomously, and actively encouraging independent decision-making (Chiniara and Bentein, 2016). This process helps team members feel valued and motivated to adapt to changing demands. Furthermore, servant leaders inherently highlight the fulfillment of followers’ needs by fostering open communication and transcending self-interest to support followers’ growth to their fullest potential (Sendjaya et al., 2019). By developing individuals to their maximum capacity, servant leaders equip team members with the knowledge, skills, and confidence to take initiative and adjust quickly as required by the team (Greenleaf, 2002). Scholars have also identified team empowerment (2015) and autonomy (Werder and Maedche, 2018) as crucial predictors of TA. In summary, SL facilitates empowerment and autonomy, thereby enhancing TA. Therefore, the hypothesis is proposed:

*H3a*: SL positively influences TA.

### 4.6 TA-PS

Agile teams exhibit a high degree of TA, and this has demonstrated positive impacts on PS through various mechanisms. Firstly, agile teams possess the ability to promptly respond to shifting priorities and changes in project scope, acknowledging change as an inherent aspect of the project lifecycle rather than resisting it (Werder, 2016). This adaptability enables them to meet evolving customer needs and align project goals accordingly. Secondly, agile teams emphasize frequent inspection and adaptation in short iterations, facilitating accelerated learning and timely course corrections (Krüger, 2023). Early identification and resolution of issues contribute to preventing escalation. Regular evaluation of progress and results through constant feedback loops enhances the likelihood of achieving PS. Thirdly, agile teams prioritize people and communications over processes and tools, fostering greater autonomy and ownership among team members (Beck et al., 2001). Bourgault et al. (2008) and Imam (2021) have supported that autonomy is a critical antecedent for PS. As a result, the agility exhibited by project teams exerts a positive influence on project outcomes. Therefore, the hypothesis is proposed:

*H3b*: TA positively influences PS.

### 4.7 The mediating role of TA

Servant leaders exhibit behaviors such as empowering followers through delegation of responsibility, encouraging autonomous decision-making, and actively supporting followers’ personal growth (Chiniara and Bentein, 2016). Team members feel valued and motivated to adapt to changing demands (Sendjaya et al., 2019), and they build confidence to take action quickly, which is required by the team (Greenleaf, 2002). By equipping team members in this way, servant leaders develop a high-level agility in the team. In turn, an agile team is better able to survive in response to shifting priorities and cope with uncertainties characterized by projects. Furthermore, TA has been empirically associated with enhanced team performance and positive outcomes (Liu et al., 2015; Werder, 2016). Agile teams outperform non-agile teams on various project outcomes, including meeting scope, schedule, and customer requirements (Conforto et al., 2014). In summary, SL enables TA by empowerment and prioritization on their growth and autonomy. In turn, agile teams achieve PS through their ability to take adaptations swiftly, learn and adjust. Therefore, the hypothesis is suggested:

*H3c*: TA plays a positive mediating role between SL and PS.

### 4.8 TLO-TA

Teams with high lever TLO exhibit openness to new ideas, and a willingness to challenge assumptions. TLO further enables a collective unit to accustom to evolving contexts, persistently refine procedures and operations, and ascertain novel and superior approaches for accomplishing team goals (Bunderson and Sutcliffe, 2003). This learning mindset has been linked to greater TA. Edmondson (1999) found that team learning behaviors fostered team flexibility and adaptability. By activating the mechanisms of SLT, positive behaviors are duplicated from servant leaders and new knowledge is disseminated among members, learning-oriented teams are better equipped to adjust strategies and meet changing demands. In rapidly evolving environments, learning teams are able to quickly perceive cues, re-evaluate assumptions, and find innovative solutions (Gong et al., 2009). They accumulate experience and insights that enable them to adjust adeptly (Jyothi and Rao, 2012). In contrast, teams fixed in their ways of thinking and operating tend to lack the agility to adapt and perform well. In summary, teams that emphasize continuous learning and growth develop the adaptability needed in dynamic contexts. Fostering TLO can positively impact TA and performance. In addition, empirical study confirms that TLO is positively associated with TA-related factors, including adaptive behaviors (Bunderson and Sutcliffe, 2003), team planning processes (Pearsall and Venkataramani, 2015), and team task reflexivity (Wang and Lei, 2018). Thus, the hypothesis is proposed:

*H4*: TLO positively influences TA.

*H5*: TLO and TA play sequential mediating roles between SL and PS.

## 5 Methods

### 5.1 Sample and procedure

The sample for this study encompassed 306 individuals engaged in construction projects in China. Participants represented diverse roles within these construction roles, including civil engineers, quantity surveyors, and MEP (Mechanical, Electrical, and Plumbing) engineers who contributed data through the survey. Data acquisition was facilitated through two avenues, including the China State Construction Association (CSCA) and the alumni network.

The data collection process was conducted using an online survey platform called Wenjuanxing^1^, a widely used professional tool in China. The initial questionnaire was designed based on the research objectives and a comprehensive literature review. The questionnaire consisted of three main parts. The first part provided an introduction to the survey, explaining its purpose, the confidentiality of responses, and instructions for completion. The second part focused on demographic questions, gathering information about the respondents’ background, such as their ages, years of experience, and educational qualifications. The third part contained the variable measurement scales, which included questions related to the key constructs of the study.

To ensure the validity and reliability of the questionnaire, a rigorous adaptation process was employed. First, the items utilized in previous relevant studies were translated from English into Chinese. Then, a group of six members, including five graduate students and one professor with expertise in construction management, carefully reviewed the questionnaire to prevent any inconsistencies. Additionally, pilot tests were conducted with a small sample of 20 respondents. The questionnaire was revised according to their feedback before being administered to the target respondents.

The process of gathering data began with the recruitment of 30 individuals who had participated in construction projects in China, each with more than 2 years of experience. To achieve a varied sample, the initial recruitment consisted of 11 quantity surveyors, 12 civil engineers, and 7 MEP engineers. These individuals were selected and invited via the CSCA and alumni associated with engineering management and cost disciplines. Following this, a snowball sampling method was utilized, wherein each initial participant forwarded the questionnaire link to other eligible participants. The questionnaire provided detailed instructions, emphasizing the confidentiality of responses. Participants were encouraged to circulate the survey among colleagues meeting the standards. The survey link was distributed through various channels, including social media and email. To ensure the uniqueness of our respondents and to prevent any individual from submitting the survey more than once, this study implemented IP address tracking and browser cookie checks. These technical safeguards effectively prevented any duplication of responses, thereby maintaining the integrity and uniqueness of our data collection process.

After around 3 months of spreading, 343 questionnaires were gathered, with 306 responses deemed valid for analysis. Thirty-seven responses were excluded based on the following criteria. Firstly, responses with a completion time of less than 120 s were discarded. This criterion was based on the average completion time observed from 15 students majoring in Engineering Management and Engineering Cost. Their educational background, which includes familiarity with industry-specific language and concepts, enables a precise evaluation of the time needed to complete the questionnaire. These students were part of a separate preliminary time trial and were not included in the final study population. Secondly, responses with identical answers across all questions were deemed uncommon and were excluded. Thirdly, illogical responses, such as reporting an age of 25 with over 12 years of experience, were also excluded. Table 1 presents the demographic information of the remaining participants.

**Table 1:** Demographics information.

Item		Frequency	Percent (%)
Gender	Male	156	50.98
	Female	150	49.02
Age	20–30	37	12.09
	30–40	107	34.97
	40–50	98	32.03
	>50	64	20.92
Education (years)	Below undergraduate	76	24.84
	Undergraduate	169	55.23
	Master and above	61	19.93
Member experience (years)	<3	5	1.63
	3–5	12	3.92
	5–10	49	16.01
	10–15	56	18.30
	>15	184	60.13
Project duration (years)	<5	65	21.24
	5–10	81	26.47
	10–15	71	23.20
	>15	89	29.08
Project member	<50	103	33.66
	50–100	16	5.23
	100–200	61	19.93
	>200	126	41.18

### 5.2 Measurement

Participants, unless otherwise specified, utilized a five-point Likert scale, ranging from 1, indicating “Strongly Disagree,” to 5, denoting “Strongly Agree.” The subsequent section delineates measurements employed in this study:

Servant leadership employed SLBS-6 from Sendjaya et al. (2019). A sample item was the following: “My project manager uses power in service to others, not for his or her ambition.” (Cronbach’s *α* = 0.887).

Team learning orientation was measured by five items adapted from Bunderson and Sutcliffe (2003). Participants in this study utilized a five-point Likert scale, ranging from 1, indicating “Very Low Extent,” to 5, denoting “Very High Extent.” A sample item was the following: “Our team looks for opportunities to develop new skills and knowledge” (Cronbach’s *α* = 0.908).

Team agility measurement utilized four items from Liu et al. (2015). A sample item is as follows: “Our team’s responsiveness to changing organizational conditions is timely.” (Cronbach’s *α* = 0.814).

Project success was measured using seven items from Aga et al. (2016). The questionnaire included items such as: “The project was completed on time.” (Cronbach’s *α* = 0.892).

In line with prior studies, the analysis incorporated demographic elements such as gender, professional expertise, age, and educational background, acknowledging their potential impact on respondents’ evaluations. Furthermore, team size and project duration were also taken into account (Barrick et al., 2007; Aga et al., 2016).

## 6 Analysis and results

RStudio Version 2023 was utilized to analyze the data. SEM techniques were utilized to evaluate the proposed model and examine the postulated assumptions. Confirmatory factor analysis (CFA) was implemented to authenticate the measurement patterns denoting the variables within the overarching structural equation model. Data Analysis employs the SEM methodology, a sophisticated statistical approach that integrates factor analysis with multiple regression analysis. This technique is adept at scrutinizing the intricate relationships between observable variables and the underlying latent constructs, all within the context of a theoretical framework. Furthermore, the application of bootstrap methods alongside SEM provides a robust mechanism for assessing the hypothesized relationships, thereby ensuring a comprehensive and rigorous examination of the conceptual framework.

### 6.1 Reliability and validity

Cronbach’s alpha (*α*) coefficients were considered to evaluate reliability and internal consistency. The value of α above 0.7 is generally considered indicative of satisfactory reliability (Vaske et al., 2017). As displayed in Table 2, the *α* coefficients for all constructs met this threshold. Composite reliability (CR) was also examined to confirm internal consistency. CR values above 0.7 are favorable (Bagozzi and Yi, 1988). The CR values for SL, TLO, TA, and PS, displayed in Table 2, all exceeded 0.7, demonstrating satisfactory internal consistency. Additionally, all items’ loadings surpassed 0.5, denoting adequate item reliability. Furthermore, the average variance extracted (AVE) for each variable is more than 0.5, as seen in Table 2, indicating that the constructs adequately captured their intended concepts (Fornell and Larcker, 1981). In addition, as shown in Table 2, the value of AVE surpassed the correlations for all constructs, suggesting satisfactory discriminant validity and that each construct measured a distinct underlying concept. Moreover, Table 3 displays the model fit indices, all of which surpass the recommended thresholds as suggested by Doğan and Özdamar (2017). The results indicated the acceptable adequacy of the model.

**Table 2:** Reliability and validity.

	Items	Item loading	CR	Cronbach’s *α*	AVE	SL	TLO	TA	PS
SL	6	0.728 ~ 0.785	0.887	0.887	0.567	**(0.753)**			
TLO	5	0.792 ~ 0.840	0.909	0.908	0.666	0.370**	**(0.816)**		
TA	4	0.681 ~ 0.800	0.816	0.814	0.527	0.253**	0.307**	**(0.726)**	
PS	7	0.681 ~ 0.793	0.892	0.892	0.542	0.482**	0.509**	0.453**	**(0.736)**

** means the correlation is significant at the 0.01 level (2-tailed); diagonal bolded values in brackets are the square root of AVE.

**Table 3:** Model fitness.

Measure	Estimate	Threshold
CMIN	235.927	–
DF	203	–
CMIN/DF	1.162	<3
CFI	0.991	>0.9
GFI	0.936	>0.9
SRMR	0.036	<0.08
RMSEA	0.023	<0.06

CMIN, Chi-square value; DF, degree of freedom; CFI, comparative fit index; GFI, goodness of fit index; SRMR, standardized root mean square residual; RMSEA, root mean squared error of approximation.

### 6.2 Common method bias

Two approaches were utilized to avoid the common method bias (CMB). First, the initial principal component elucidated 34.847% of total variance, falling under the 50% threshold indicative of substantial CMB (Podsakoff et al., 2003). In addition, the proposed model demonstrated a significantly improved fit from the single-factor model (Δ*χ*^2^ = 1354.626, Δdf = 6, *p* < 0.001), providing evidence against CMB (Guo et al., 2016). As shown in Table 4.

**Table 4:** Measurement model fit indices.

Model	*χ* ^2^	df	*χ*^2^/df	CFI	TLI	RMSEA
proposed model (TFL, AT, CT, PS)	235.927	203.000	1.162	0.991	0.989	0.023
Single-factor model (TFL + AT+CT + PS)	1590.553	209.000	7.610	0.604	0.562	0.147

### 6.3 Hypothesis testing

This study employed the Bootstrap method with 5,000 samples to conduct path analysis and assess hypotheses. Direct effects of the model are summarized in Table 5, with a detailed breakdown provided subsequently. Table 5 outlines the outcomes for Hypotheses 1, 2a, 2b, 3a, 3b, and 4. These results reveal significant support for Hypotheses 1 (*β* = 0.224, *p* < 0.05), 2a (*β* = 0.498, *p* < 0.001), 2b (*β* = 0.361, *p* < 0.001), 3a (*β* = 0.224, p < 0.05), 3b (*β* = 0.392, *p* < 0.001), and 4 (*β* = 0.233, *p* < 0.05), indicating their statistical significance. Additionally, our analysis reveals that none of the control variables were validated. As shown in Table 5.

**Table 5:** Structural model results.

Hypotheses	Proposed effect	Estimate	S.E.	*p*-value	Results
H1: SL-PS	+	0.224*	0.079	0.005	Supported
H2a: SL-TLO	+	0.498**	0.087	0.000	Supported
H2b: TLO-PS	+	0.361**	0.060	0.000	Supported
H3a: SL-TA	+	0.224*	0.079	0.005	Supported
H3b: TA-PS	+	0.392**	0.079	0.000	Supported
H4: TLO-TA	+	0.233*	0.075	0.002	Supported
Control variable					
Gender		0.037	0.080	0.643	Not significant
Age		0.024	0.029	0.398	Not significant
Education		−0.004	0.066	0.948	Not significant
Member experience		−0.019	0.029	0.510	Not significant
Project duration		−0.002	0.006	0.789	Not significant
Project members		−0.000	0.000	0.321	Not significant

**p < 0.001, *p < 0.05.

Table 6 shows the endorsement of Hypothesis 2c, suggesting the mediating effect of TLO between SL and PS. The 95% confidence interval (CI) for the coefficients (0.096, 0.279) excludes zero, thus affirming TLO’s mediating role between SL and PS. Similarly, Hypothesis 3c, proposing the mediation of TA is demonstrated. Examination indicates that the 95% CI for the coefficients (0.022, 0.174) does not encompass zero, thereby confirming TA’s mediating role between SL and PS. Furthermore, Hypothesis 5, which postulates the sequential mediating effects of TLO and TA, is supported. The 95% CI (0.015, 0.086) does not include zero, showing this sequential mediation.

**Table 6:** Analysis of mediating effects.

Hypotheses	Proposed effect	Relationship	Estimates	S.E.	*p*-value	Boot 95%CI	Results
H2c	+	SL-TLO-PS	0.180**	0.047	0.000	[0.096,0.279]	Supported
H3c	+	SL-TA-PS	0.088*	0.039	0.025	[0.022,0.174]	Supported
H5	+	SL-TLO-TA-PS	0.045*	0.018	0.011	[0.015,0.086]	Supported
Total effect			0.313**	0.064	0.000	[0.196,0.443]	

Bootstrap sample size = 5,000 times; **p < 0.001, *p < 0.05.

## 7 Discussion

Based on SLT, this study examined the direct and indirect relationships between SL and PS in Chinese construction projects. As predicted, a positive correlation between SL and PS was found. This finding confirms the notion that leadership is a fundamental factor for PS (Ellahi et al., 2022). Moreover, this finding is aligned with previous studies but in different contexts. For instance, Ellahi et al. (2022) found this relationship in the context of software projects, while Malik et al. (2022) demonstrated this relationship in non-governmental organizations, and Nauman et al. (2022b) supported this relationship in vocational training organizations. However, these findings were primarily confirmed in eastern countries like Pakistan. As suggested by Zhang et al. (2021), the relationships between SL and its outcomes are moderated by cultural factors such as traditionality. Both Pakistan and China are Eastern societies that emphasize collectivistic values (Hofstede, 2001; Basabe and Ros, 2005). SL is likely to exhibit more positive effects in a collectivistic cultural environment. Collectivism values cooperation, mutual respect, and concern for others, which aligns with the core tenets of SL (Sendjaya and Pekerti, 2010). In collectivistic cultures, people tend to prioritize the collective interests of the team over individual goals. Servant leaders, by exemplifying service, and empowering team members, can enhance team cohesion, which is conducive to collaboration among project members and, consequently, to achieve PS (Kyriazis et al., 2017). Thus, it is essential to note that the effects of SL may vary across different cultures (Eva et al., 2019). Future research is encouraged to investigate the effects of SL across diverse cultural contexts.

Another finding of this study was the positive connection between TLO and PS in Chinese construction projects. This finding confirms the conclusion of Bunderson and Sutcliffe (2003) that team focus on learning can yield positive outcomes for team effectiveness. It also provides supportive evidence that TLO can promote favorable team outcomes, aligning with prior studies that have positioned TLO as an antecedent of team performance indicators, such as employee creativity (Qian and Kee, 2023) and team goal mental models and team planning processes (Pearsall and Venkataramani, 2015). The significance of TLO stems from its ability to motivate members to participate in various learning activities, thereby enhancing team effectiveness (Chiu et al., 2021). In the dynamic and complex environment of construction projects, where teams frequently encounter challenges and uncertainties, a learning-oriented mindset can facilitate the acquisition, sharing, and application of new knowledge and skills, enabling teams to respond proactively to changes and contribute to PS.

Additionally, TA was found to have a positive relationship with PS in Chinese construction projects. This finding supports the existing literature that highlights the importance of TA in project management (Conforto and Amaral, 2016). Conforto et al. (2014) contend that the existence of specific enablers for agile project management indicates the potential to extend the application of agile project management theories and practices beyond the software industry. The present study provides empirical evidence supporting the generalization of agile methods to industries other than software, such as the construction sector. Moreover, this finding suggests that TA is particularly critical in the Chinese construction context, where projects often involve intricate coordination among multiple stakeholders, complex regulatory frameworks, and fluctuating market conditions (Tang et al., 2012). Agile teams can navigate these complexities more effectively, mitigating potential risks and capitalizing on favorable circumstances. Furthermore, the study by Liu et al. (2015) confirms that collectivism, a prominent cultural value in China, is conducive to TA. The emphasis on group cohesion, coordination, and responsiveness to environmental changes in collectivistic cultures aligns well with the context of Chinese construction projects.

Next, another finding is the positive mediation of TLO between SL and PS. It confirms the importance of team learning processes in translating the influence of leadership styles into improved team and organizational outcomes (Bucic et al., 2010). Moreover, the results also support the argument that person-focused leaders foster team learning (Koeslag-Kreunen et al., 2018). Specifically, our results indicate that SL fosters a learning-oriented mindset within teams, characterized by a shared commitment to continuous learning, knowledge sharing, and embracing challenges as opportunities for growth (Liden et al., 2014). This collective learning orientation, in turn, contributes to project goals and objectives, acting as a critical mediating mechanism linking SL and PS.

Furthermore, TA was also found to be a positive mediator between SL and PS. This finding is consistent with previous studies that have emphasized the importance of TA in enhancing team effectiveness and performance, particularly in dynamic and rapidly changing environments (Werder, 2016). In the context of construction projects, where teams often face intricate coordination challenges, complex regulatory frameworks, and fluctuating market conditions, the capability to swiftly adapt to changes and respond effectively to unforeseen circumstances is paramount. Servant leaders, by prioritizing the growth of team members, create a climate that fosters trust, empowerment, and humility (Sousa and Van Dierendonck, 2016; Lee et al., 2020). This supportive climate enables teams to develop a heightened sense of agility, allowing them to reconfigure resources, adjust strategies, and coordinate actions efficiently in response to emerging challenges or opportunities, which in turn, engender PS.

Finally, perhaps most importantly, this is the first study finding that TLO and TA play sequential mediating effects between SL and PS. Despite TA is important for PS, both parallel and sequential mediation analyses revealed that the indirect effect through TLO is critically significant. In other words, compared to the enhancement of TA, the facilitation of TLO is a relatively more efficacious mechanism through which SL improves PS. This finding emphasizes the vital role of TLO, especially in agile teams. As Edmondson (1999) suggests, a team’s learning orientation is a fundamental enabler for agility, as it promotes team flexibility which is essential for teams to rapidly adapt to changing environments. The critical importance of TLO in mediating the SL-PS relationship could be attributed to the fact that a learning-oriented mindset not only facilitates the TA but also nurtures the ability to achieving PS. A team’s collective commitment to learning enables it to proactively identify and address challenges, adapt to changing circumstances, and leverage collective knowledge and expertise to drive project outcomes.

### 7.1 Theoretical implication

The findings offer several theoretical implications. First, the positive connection between TLO and PS in the context of construction projects extends the understanding of the role of team learning processes in driving project outcomes. This finding is in line with previous studies that have positioned TLO as an antecedent of team performance (Bunderson and Sutcliffe, 2003; Wang and Lei, 2018), and further generalizes this relationship to construction projects.

Second, the positive relationship between TA and PS in the construction project context aligns with existing literature that highlights the importance of TA in enhancing team effectiveness and performance, particularly in dynamic environments (Krüger, 2023). The study provides empirical evidence supporting the generalization of agile methods and practices beyond the software industry, extending their application to the construction sector.

Finally, the paper is the first study to demonstrate the sequential mediations of TLO and TA between SL and PS. This novel finding extends our understanding of the complex mechanisms through which SL influences project outcomes. The parallel mediating mechanisms of TLO and TA indicate that servant leaders can foster both a learning-oriented mindset and TA simultaneously, which in turn contribute to PS through distinct yet complementary pathways. This finding highlights the multifaceted nature of SL and its ability to positively influence multiple team processes concurrently, ultimately leading to improved project performance. In addition, the sequential mediating effects of TLO and TA provide insights into the potential sequential effects of SL on team processes and project outcomes. Specifically, this finding suggests that servant leaders may first nurture a learning-oriented mindset within teams, which then facilitates the development of TA, ultimately leading to enhanced PS. This sequential model offers a nuanced understanding of the relationships among leadership, team processes, and project performance, and suggests a potential causal chain through which SL exerts its influence.

### 7.2 Practical implication

This study also provides several practical implications. Firstly, the findings underscore the potential of SL to cultivate TLO and TA, ultimately contributing to PS. The identification of SL as a catalyst for TLO and TA suggests that organizations and project leaders can enhance project outcomes by adopting and promoting SL behaviors within their teams. Given that project managers often coordinate members with diverse specialties and cultures (Bell and Kozlowski, 2002), SL appears particularly well-suited for this scenario. SL, characterized by a leader’s emphasis on serving others and facilitating their growth, aligns with the dynamic where team members possess specialized expertise. In such contexts, the project manager can leverage SL principles to foster a supportive and empowering environment, allowing team members to thrive and contribute their expertise fully. Therefore, embracing SL practices can lead to more effective project management, better utilization of team members’ skills, and improved overall project outcomes.

Additionally, the study highlights the importance of recognizing TA as a critical mediator between SL and PS. This finding underscores the need for project managers and organizations to actively foster TA within their project teams. Agile teams possess the ability to swiftly adapt to changes, reconfigure resources, and coordinate actions effectively, which is crucial for navigating the complexities and uncertainties inherent in project environments. By promoting practices that enhance TA, such as empowerment, cross-functional collaboration, and rapid decision-making, project managers can increase the likelihood of achieving PS. Moreover, it is crucial for project teams to reach a balance between agility and regulated processes. Project managers should establish clear guidelines and frameworks that provide structure while allowing for flexibility and adaptability. This balance can help harness the benefits of TA while ensuring adherence to project standards and best practices.

Furthermore, understanding the sequential mediating effects of TLO and TA between SL and PS offers a nuanced perspective for practitioners. The sequential mediating roles can inform the alignment of team processes with different project phases. For instance, during the initial stages of a project, emphasis can be placed on cultivating a learning-oriented mindset, while later stages may prioritize agile practices and rapid adaptation as the project progresses and evolves.

### 7.3 Limitations

This study possesses several limitations. Firstly, the use of cross-sectional data impedes the establishment of causal relationships. The observed correlations among SL, TLO, TA, and PS do not necessarily indicate a causal relationship. Future studies could embrace longitudinal data, enabling a more comprehensive examination. Secondly, using a single respondent to complete the questionnaire may introduce CMB. This bias occurs when measurements of both predictor and criterion variables are obtained from the same source, which can potentially influence the proposed relationships among the variables (Podsakoff and Organ, 1986). However, two methods were employed to assess CMB, and the results indicate that CMB may not be a significant concern. The final limitation pertains to the single-country data collection. Cultural influences may impact the generalizability of these empirical findings. Future studies are encouraged to collect data across multiple countries, especially those with diverse cultural backgrounds.

## 8 Conclusion

This study sought to explore the mediating effects of TLO and TA between SL and PS in Chinese construction projects. The results show that TLO and TA act as both parallel and sequential mediators between SL and PS. The findings offer valuable insights for practitioners to achieve PS, emphasizing the importance of cultivating a learning-oriented mindset, promoting TA, and developing SL, particularly in Chinese construction projects.

## Data availability statement

The raw data supporting the conclusions of this article will be made available by the authors, without undue reservation.

## Ethics statement

The studies involving humans were approved by Institutional Review Board of SolBridge International School of Business. The studies were conducted in accordance with the local legislation and institutional requirements. The ethics committee/institutional review board waived the requirement of written informed consent for participation from the participants or the participants’ legal guardians/next of kin because details about the study purpose and participant rights were clearly stated in the distributed questionnaire.

## Author contributions

HH: Writing – original draft, Validation, Supervision, Software, Resources, Methodology, Investigation, Funding acquisition, Formal analysis, Data curation, Conceptualization. XZ: Writing – review & editing, Visualization, Investigation.

## Footnotes

^1^ https://www.wjx.cn/
